# A FEM-Experimental Approach for the Development of a Conceptual Linear Actuator Based on Tendril's Free Coiling

**DOI:** 10.1155/2017/6450949

**Published:** 2017-07-25

**Authors:** Luca Cortese, Selena Milanovic, Renato Vidoni

**Affiliations:** ^1^Faculty of Science and Technology, Free University of Bozen-Bolzano, Piazza Università 5, 39100 Bolzano, Italy; ^2^Department of Mechanical and Aerospace Engineering, Sapienza University of Rome, Via Eudossiana 18, 00184, Rome, Italy

## Abstract

Within the vastness of the plant species, certain living systems show tendril structures whose motion is of particular interest for biomimetic engineers. Tendrils sense and coil around suitable grips, and by shortening in length, they erect the remaining plant body. To achieve contraction, tendrils rotate along their main axis and shift from a linear to a double-spring geometry. This phenomenon is denoted as the free-coiling phase. In this work, with the aim of understanding the fundamentals of the mechanics behind the free coiling, a reverse-engineering approach based on the finite element method was firstly applied. The model consisted of an elongated cylinder with suitable material properties, boundary, and loading conditions, in order to reproduce the kinematics of the tendril. The simulation succeeded in mimicking coiling faithfully and was therefore used to validate a tentative linear actuator model based on the plant's working principle. More in detail, exploiting shape memory alloy materials to obtain large reversible deformations, the main tendril features were implemented into a nickel-titanium spring-based testing model. The results of the experimental tests confirmed the feasibility of the idea in terms of both functioning principles and actual performance. It can be concluded that the final set-up can be used as a base for a prototype design of a new kind of a linear actuator.

## 1. Introduction

Climbing plants can be a meaningful source of inspiration for biomimetic purposes for their intriguing complexity and functional perspectives [1]. Among these, climbing tendril-bearer plants show an interesting way to search, grasp, and climb a support [2–4], a behavior that can be studied and implemented in future bio-inspired technologies [5–8]. Indeed, by means of tendrils, that is, filiform, irritable, and long organs, they are able to coil around a support and grasp it, allowing the plant to gain vertical displacement. As it is known, tendrils describe three main movements [3, 9–16]:

- Circumnutation: an endogenous movement that increases the probability of contact with supports
- Contact coiling: during which the stimulated tendril coils around a support
- Free coiling: throughout which the tendril develops helical coils along its axis, not necessarily as a result of stimulation.

As a whole, the study has been divided into two sections: the first presents a model and FEA of the tendril's free-coiling phase and the second investigates the efficiency of the tendril's geometry in lifting weights. Hence, the core lies in analyzing the effects of its geometry on its range of motion, rather than in justifying its internal structure. Indeed, starting from a linear configuration, the tendril's geometry evolves into two helices with opposite rotational directions. The rotation around its axial axis is denoted as free coiling and begins after the tendril has completed the phase of contact coiling. This final change in geometry results in a contraction, therefore lifting the remaining plant body. The fundamentals for this behavioral analysis have been extracted from results available in literature (e.g., [8, 17]).

Moreover, this pulling movement is achieved by creating an elastic spring-like connection between the stem and the grasped support, able to resist to highly stressed conditions such as wind and loads [3].

**Figure 1:**
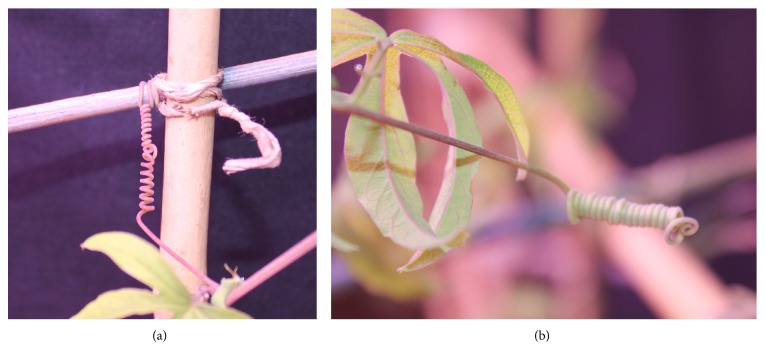
Tendril free coiling: (a) grasped tendril; (b) nongrasped tendril.

Recent biological studies [17] have shown that the tendril body is partially made of a specific kind of cells named G-cells (or G-fibers). These are typically found in trees and are those which dehydrate during the growth process granting stiffness to the plant's body. Indeed, during the free-coiling process, the entire structure dehydrates, including the G-fibers, and thus becomes more rigid, preventing uncoiling. In general, lignification seems to be highest in the fibers closest to the touching surface [15]. This spring spiral structure has very often been compared to a classic telephone cord and might be described by an ideal helical spring. Darwin [3] observed that the same numbers of spirals are created in both directions (i.e., clockwise and counterclockwise) resulting in a zero twist on the axis, see Figure 1(a). The segment of spiral inversion which unifies the two helices is called tendril perversion. If no grasping occurs, the tendril curves and creates a simple spiral; that is, coiling occurs in one direction only, see Figure 1(b).

For tendrils, a proper stiffness is a very important factor since the plant has to find the right compromise to provide stability and at the same time to be able to withstand strong winds, for example, by bending. It appears that the number of coils and the radius of curvature of the coil play a major role. In previously conducted studies [8, 18–21], the mechanical behavior of springs and perversion was evaluated. This was necessary in order to understand the influence that various geometrical factors have on the tendril behavior. As a result, it seems that tendrils tend to create coils with a very small diameter since by decreasing the diameter, the stiffness of the spring increases. The number of coils that the tendril forms is also of great importance; in fact, with a decreasing number of coils, the total stiffness increases. Other studies [8, 17] have also investigated factors such as the age of the tendril and what is the influence of the perversion on a force-versus-length relationship.

It was found that for the same variation of length, the force of the spring almost doubles in old tendrils. For what concerns the perversion instead, it appears that the variation in length of a tendril with perversion is twice the one that has none. By combining these factors, it is possible to design a spring corresponding to specific needs.

Even if these results explain the geometry and the stiffness behavior of the tendril, it is of fundamental importance to be able to understand how to replicate the plant's free-coiling and perversion creation, the stiffness given by the G-fiber dehydration, and the forces applied that act on the filiform organ in order to provoke the free-coiling phenomenon.

To the authors' knowledge, even if, from the technological point of view, some robots and prototypes able to bend, contract, and extend have been developed (e.g., serpentine robots [22], continuum tendril manipulators [23–26], and smart springs [27]), only few physical emulators of the free coiling can be found in literature. Among these, remarkable examples of materials that can autonomously change their shape in response to external stimuli are the soft actuator based on a dual-layer dual-composition polysiloxane-based liquid crystal [28], the fiber actuators driven by solvent and vapour stimuli [29] and the synthetic self-shaping materials [30]. However, a structured analysis and evaluation of both the elastomechanical behavior and replication for bio-inspired engineering purposes are still unavailable.

Given the previous considerations, this study aims firstly at investigating and explaining the fundamentals of the mechanics behind the free-coiling and perversion creation by means of a reverse-engineering approach, using the finite element method (FEM) as in [31], see Section 2. This was achieved by borrowing structural models and FE methodologies normally used in mechanics and adapting them to simulate the biological “tendril system.”

At this point, it is essential to underline that the primary aim is designing a model whose free-climbing motion (i.e., from linear to double-helix geometry) emulates the one of the tendril. This being said, the means to achieve this result are not bound to the biological constraints of the plant. Indeed, the properties of the materials (e.g., Young's modulus) used in the model do not correspond to ones of the plant.

Three different models were devised: the first simulated the G-cell structure only, reproducing bending, twisting, and perversion; the second tried to emulate the behavior of the whole tendril, comprising the G-cells embedded in the green body; and the last model focused on a tentative study of a linear actuator in which the actuation is provided by a tendril-like mechanism.

Secondly, in Section 3, the functioning principles of the free-coiled shape are studied with the aid of a testing model based on smart memory alloy (SMA) springs. SMAs were used being suitable to produce (by issuing an appropriate electric command) the significant displacement required by an effective actuator. To this purpose, a mechanical system that implements the “features” of the tendril (i.e., elastic spring-like behavior, perversion element) as a linear actuator was set up. Experiments were performed to check both the effectiveness and performance of the mechanism.

Finally, Section 4 reports the discussion and the conclusions on the achieved results.

## 2. Understanding the Free-Coiling Phase: A Finite Element Approach

**Figure 2:**
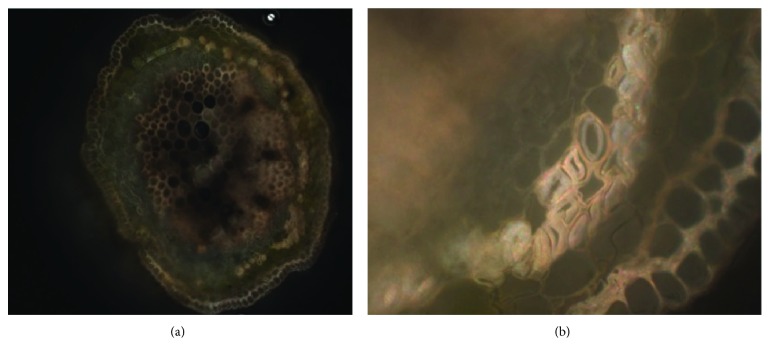
A modified picture from [17]. (a) Cross-sectional area of a *Passiflora* tendril; (b) magnification of the lateral wall of the tendril section. The G-cells can be distinguished from the remaining plant body through the thick delineation of their oval cell structure.

With the aim of designing the working principle for the tendril-based linear actuator, an in-depth biological study of the tendril nature appeared to be fundamental. Looking at its internal structure, it can be observed that a section closer to the edge is made of two dissimilar layers of G-cells [17] (Figure 2). The same kind of cells can be found in various plants that dehydrate partially in order to provide stability to their stem or trunk. The function of the G-cells is initiated with the end of the grasping phase. The assumption that the authors want to prove is that two concurrent mechanisms contribute to the helix-like structure:

- The lignification of the cells induces the bending of the tendril due to a differential contraction of the two different layers of G-cells.
- A drag force originates at the bottom end of the tendril during contraction, caused by the weight of the plant that is being lifted; its direction differs from the axis of the tendril dependent on the position of the remaining plant body. This force is responsible for triggering coiling along bending.

Additionally, being constrained at both ends (i.e., grasping point on one side and stem on the other), the lignification process leads to the formation of two helices unified by a perversion. The rotational movement of the plant is therefore limited around its axis.

### 2.1. FEM Model of the G-Cells

Finite element analysis was employed to validate the suppositions on the actual free-coiling mechanisms. The first approach consisted of isolating the G-cells from the rest of the tendril body and simulating their coiling effect only. This is to prove the idea that they could be the driving elements of the entire process while the remaining part of the body just moves according to them.

**Figure 3:**
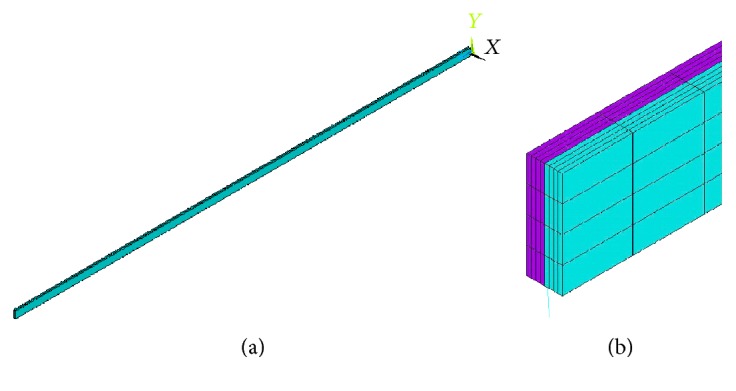
(a) Three-dimensional model simulating the G-fibers. (b) Detailed view of the two bonded stripes.

The geometry of the G-cells was emulated by modeling two thin stripes bonded together, each having a thin rectangular cross section (Figure 3). To mimic the real plant attitude, as supposed, the layers had to present a different level of contraction to provoke bending or even to maximize the bending effect itself; one layer had to be stretched while the other had to be compressed. Such behavior was achieved by means of a “fictitious” thermo-structural analysis, issued to obtain the desired shape through a differential deformation of the two stripes. Basically, the irreversible deformation due to dehydration was emulated using thermally induced elastoplastic deformation. The structure was modelled using the actual dimensions of the tendril, and a thermal gradient was applied between the opposite larger external surfaces of the bonded stripes, possibly selecting also different thermal expansion coefficients for the two parts to further enhance deformation. Analytically, the induced bending strain, or the corresponding radius of curvature, is related to the thermal quantities through the simple expression:

(1) $$\begin{array}{c} \varepsilon \left(y\right)=\frac{y}{R}=\alpha \Delta T, \end{array}$$

where *R* is the radius of curvature, *α* is the material thermal expansion coefficient, ∆*T* is the temperature variation, and *ε* is the axial strain expressed as a function of the distance *y* from the neutral bending axis. Elastoplastic material properties were applied to emulate the irreversible lignification process. A bilinear simplified plasticity behavior was adopted providing, along with elastic constants, also the yield strength and the tangent modulus (slope) of the hardened part (Table 1). FE nonlinear analysis capabilities were activated, to describe the finite large deformation of the coils. Then, in order to take into account the force exerted by the plant body, a force was applied on the plant side end of the tendril. This force was supposed to increase in time, being related to the tendril contracting length; hence, it was induced using a spring element with adequate stiffness, fixed at one end and connected at the tendril end at the other, at a reasonable angle with respect to the tendril axis. This angle was derived from observations of the real plant geometry and movement. Great attention was paid to model all significant geometrical aspects, boundary conditions, and loads. No rotational and translational displacement along the axial direction was allowed to the tendril end grasping to the support.

Finally, a sensitivity analysis proved that an accurate solution was achieved with the entire model fine-meshed using 3200 brick elements.

Unfortunately, it was not possible to have an accurate information on the real organic behavior of the different parts of the tendril and to use them in the analysis. This would have involved dedicated and complex testing, as well as the use of more sophisticated constitutive models to account for anisotropic and inhomogeneous characteristics. Therefore, the proper material properties that the simulated G-cells should be made of, as well as the *α* and ∆*T* values, were identified to reproduce at best the real plant behavior. Figure 3 and Table 1 show the best fit geometry, dimensions, and parameters of the tuned model. It must be underlined that some of these values are physically different from the ones of the plant as well as of engineering materials and are intended only to reproduce the free-coiling deformation. This was necessary to let the numerical material models suited for metals to behave like an organic G-cell “material.”

**Figure 4:**
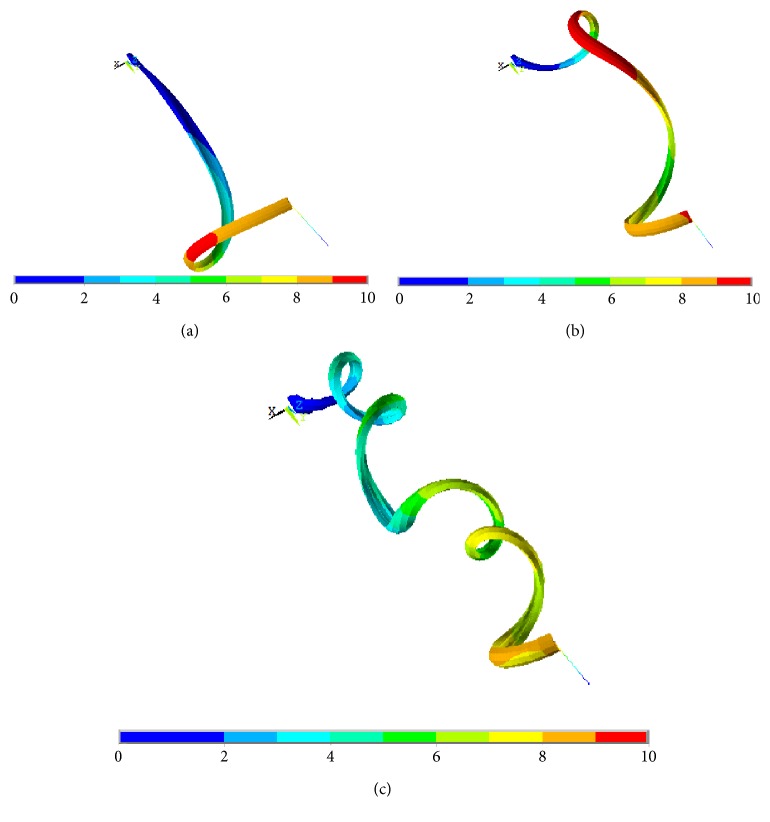
Free coiling of the G-cell model, exemplifying total displacement plots: at (a) 32%, (b) 52%, and (c) the final stage of the analysis. The underlying color bar indicates the overall displacement, in mm, ranging from blue to red as the movement of the section increases.

The numerical results demonstrated that the simulation was capable of reproducing the free-coiling kinematics reasonably.  Figure 4 presents three substeps of the free-coiling phenomenon as simulated. In the last representation, the perversion is clearly visible while in the others, the progressive bending and twisting of the structure can be observed. The formation of the perversion is correctly induced by the constraints applied on both ends and appears roughly at half of the tendril length. It represents the meeting point of the two helices with opposite rotational directions. Furthermore, it appears that the increase in the thermal coefficient causes an increase in the number of the formed coils, which can be therefore controlled through this parameter.

### 2.2. FEM Model of the Entire Tendril Body

**Figure 5:**
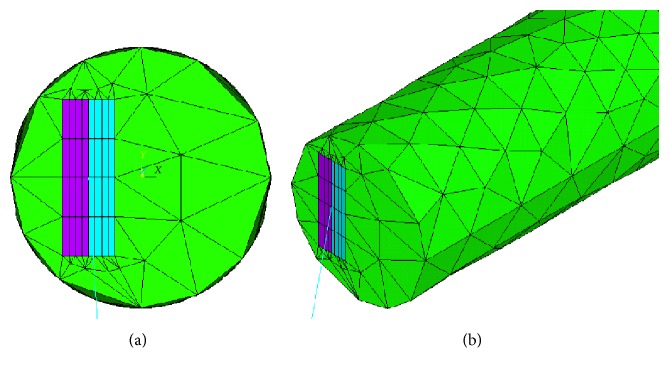
Simplified model of the tendril's internal structure. (a) Cross-sectional area of the tendril body with the G-cell section close to the left lateral wall; (b) isometric view of the 3D model of the entire tendril body, with the spring element used to apply the force exerted by the plant body.

In order to set up a simulation of the full tendril, further research was needed to include the “passive” part of the plant into the previous G-cell free-coiling simulation. Consequently, the G-fibers were positioned on one side of the tendril and enclosed in a cylinder-like envelope simulating the green part of the tendril itself, in accordance to what is observed in Figure 2.  Figure 5 shows the actual model. The geometry of the meshed elements consisted of bricks and tetrahedra, for the G-cells and the remaining tendril body, respectively.

The choice of the material of the green body played a significant role here. By assigning this part of the tendril the appropriate elastic properties, it was possible to mimic the coiling phenomenon with the G-cells being the active section and the envelope being the passive one. To grant high elasticity to the body surrounding the G-cells, which undergoes very large deformations without lignification, a hyperelastic material model (the widely used one proposed by Mooney and Rivlin) was adopted. The model was again successfully tuned to trigger the coiling phenomenon correctly. All material constants relative to this second model are reported in Table 2.

**Figure 6:**
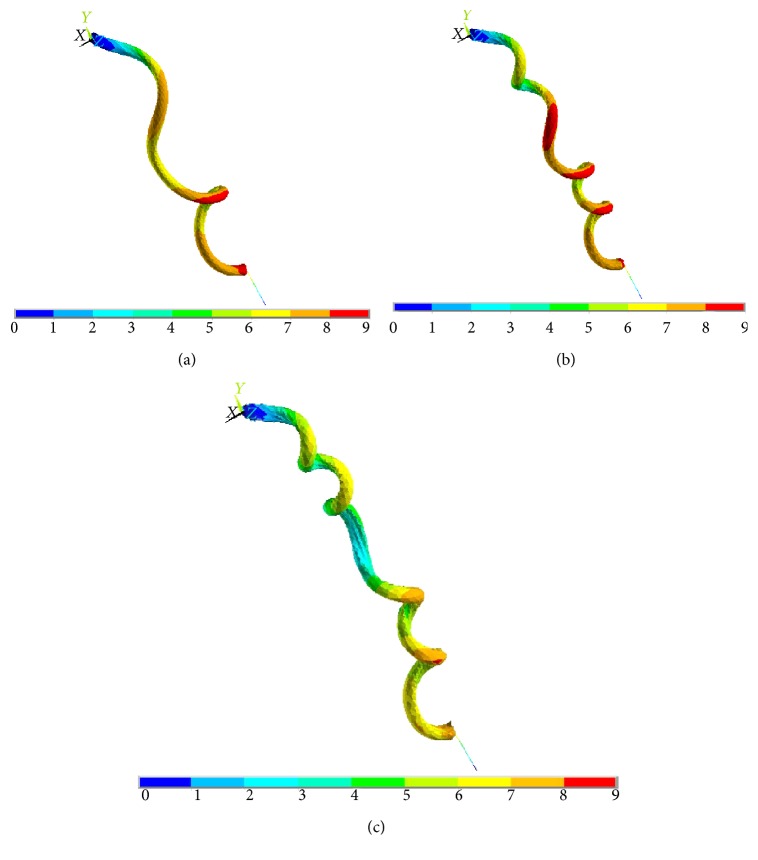
Free coiling of the entire tendril model, exemplifying total displacement plots: at (a) 32%, (b) 52%, and (c) the final stage. The underlying color bar indicates the overall displacements, in mm.

It can be observed that the application of the cylindrical envelope representing the tendril body, though less stiff than the G-cells made partially, has an influence on the final behavior (Figure 6): under the same loading conditions, the complete tendril model showed less pronounced coils (and also less coils) than the G-cell-only model. Causes of this effect are several. On the one hand, the force applied on the structure end is now also partially involved in dragging the red body such that its twisting effect is therefore reduced. Secondly, the increase in the amount of material acts as a resistant factor which partially inhibits coiling.

### 2.3. FEM Model of a Tendril-Inspired Linear Actuator

With the aim of investigating and exploring the feasibility of a tendril-inspired linear actuator, an additional simulation was devised. In this case, the major obstacle consisted of performing the analysis by applying the force acting on one end of the tendril along the actuation axis, instead of being directed out of the plane as before (as in the real plant). To achieve this, the boundary conditions were modified starting from the models previously described: on one side, all the degrees of freedom (DoFs) were constrained while on the other side, only the movement along the actuation axis was allowed. The force promoting the free-coiling effect, applied at the free translational DoF of the tendril end, was distributed uniformly on the entire cross-sectional area. The last modeling step of this analysis was necessary to induce twisting as desired, consisting of introducing a minor irregularity in the motion of the tendril. Indeed, a rotation of 0.5 degrees was applied on the nonfixed end of the tendril in order to facilitate coiling. This was necessary because the force which helped twist no longer acts out of axis as before, in the linear actuator. In an actual system, this minor rotation could be introduced on purpose to facilitate twisting. The new parameters of the linear actuator model are finally provided in Table 3.

**Figure 7:**
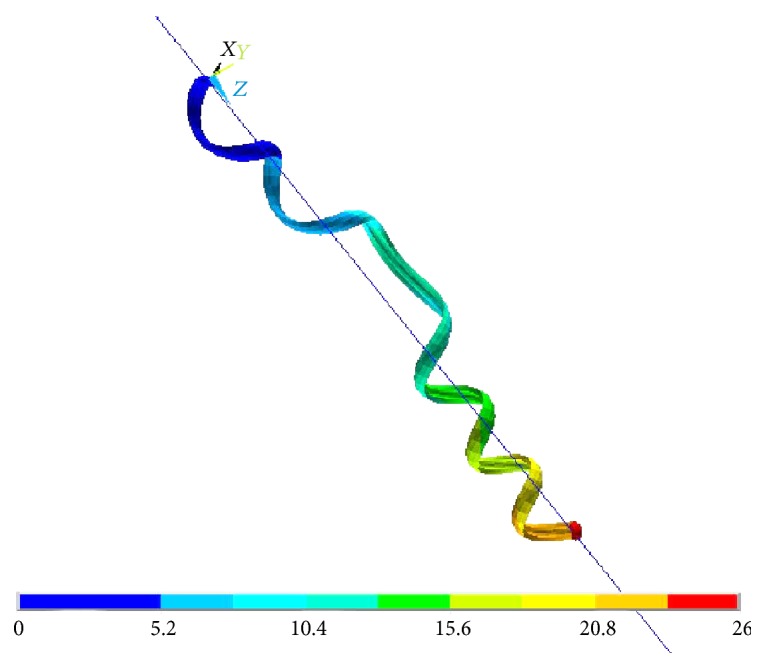
FEM analysis of a free-coiling tendril-inspired linear actuator, exemplifying the total displacement plot. The perversion is present at about half of the length of the contracted body. The line in the figure represents the linear path along which the tendril contracts. The underlying color bar indicates the overall displacements, in mm.

Figure 7 shows the output of this last simulation. A line is plotted to help the visualization of the axis and of the linear movement of the tendril body. As the main result, it can be stated that the free-coiling “mechanism,” constrained to a linear axis, is nevertheless present and still manifests its peculiar features (i.e., generation of coils, perversion, and contraction). Moreover, confirming one of the key features of the natural tendril, torsion is not present at the simulated tendril linear actuator ends, given the balancing effect of the two halves connected by means of the perversion. It seems therefore possible to exploit free coiling advantageously, to design an actuator of a mechanical system.

## 3. Experimental Model of a Tendril-Like Linear Actuator

The FE analysis of the previous section demonstrated to be effective in providing an understanding of the deformation mechanisms of the tendril and in suggesting a possible utilization of the underlying functioning principles. Nevertheless, a thermally induced actuation mechanism (besides relying on adapted material constants) would not be practical as a real-world application. Hence, to replicate the tendril model in an actual testing structure (prior to thinking at a true prototype), a feasible solution based on similar principles highlighted by the FEM evaluation had to be individuated. Eventually, the authors found a promising choice in the use of SMA springs [32]. SMAs have been constantly present in various engineering fields for the past few years, because of their low maintenance cost, corrosion resistance, and lightweight structure [33, 34]. The capability of this material relies on being able to “remember” the shape that has been imposed to it and return to it, even after strong deformations, with the application of specific stimuli (e.g., mechanical or thermal). In fact, being triggered with an appropriate electric stimulus, the SMAs react in a way that it will be proven to be suitable for the scope. In the following, proper SMA springs made of nickel-titanium (Niti) are used to devise and study a linear actuator inspired by the tendril's free-coiling effect, by following the approach in [6, 8] and by taking advantage of the suggestions and findings from the previous simulations.

More in details, as observed, in the advanced free-coiling phase, the tendril was considered at a global level two parts which contract and do not rotate between each other due to the presence of a peculiar joining element (the perversion) and the constraints at the ends. The typical mechanical element which contracts allowing large deformations may be a spring. To obtain a spring-like contraction starting from the inside of the material (this is crucial) as in the tendril, SMA springs can be profitably used and were therefore selected. In addition, a linear spring behavior, though different from the nonlinear one of the real tendril, might represent a future advantage in controlling a real actuator. Such experimental model can be designed to investigate the effectiveness of the double helical shape of the free-coiled tendril. Once more, the relevance of the geometrical appearance is put in foreground, and possible discordances with the internal structure of the actual tendril are neglected.

### 3.1. Design of the Testing Structure

**Figure 8:**
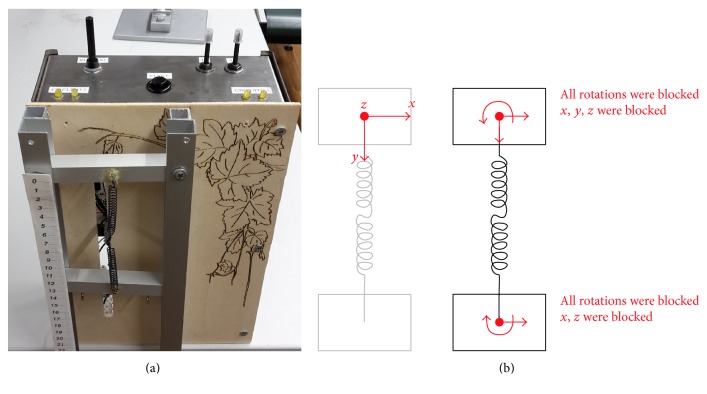
(a) Testing system: two SMA springs connected by a perversion element at one extremity and constrained on the other as highlighted in the (b) schematic representation of free and constrained DoFs.

The experimental model is composed of two NiTi springs, with opposite rotational directions, assembled in series and unified by a linear segment representing the perversion. As previously stated, in such a manner, a simplified version of the tendril where the perversion and the helices are already present was reproduced. The system was constrained at one end to a fixed crosshead of a rigid frame, while, on the other end, a vertical constant load (weight) was applied to a moving crosshead. Torsion at the extremities was visually evaluated while the axial displacement of the moving crosshead was measured through a rigid ruler fixed on the frame. The diameter of the springs and of their wire measure is respectively 6 mm and 0.35 mm. Their resistivity is 82 *μ*Ohm/cm at the high-temperature state (76 *μ*Ohm/cm at the low-temperature stage). To stimulate SMA springs with an electrical current, a piloting circuit was designed and tuned in order to supply the proper amount of electricity. In particular, the circuit needed to provide about 2 A to actuate the springs with the chosen characteristics. The layout of the arrangement is shown in Figure 8(a). On one side, the two SMA springs are connected together and, on the other side, are constrained in their motion in order to mimic the real tendril condition. The upper constraint representing the contact-coiling point of the tendril limits all DoFs, that is, the aluminium rod is blocked. The constraint at the bottom allows a translation movement only (i.e., elongation and contraction). A graphical illustration of the constrained and free degrees of freedom is given in Figure 8(b).

Different tests were devised in order to check the potential of such mechanism to mimic the behavior of the tendril. SMA springs were mounted with an opposite twisting direction, and in the first phase (i.e., test I), different stimulations were applied to estimate the torsional effect at the perversion level and at the blocked extremities.

In the second phase (i.e., test II), the contraction length of the SMA springs and the applied loads were measured during the experiments, to evaluate the stiffness of the system. The applied testing procedure for test II can be summarized in the following steps, which were repeated for different loads:

- Application of the load at the bottom rod end (range from 20 g to 200 g)
- Stimulation with an electric current (2 A, 12.5 V) to induce the contraction of the SMA springs
- Measurement of the system displacement
- Pausing for 180 s in order to allow the springs to cool down (i.e., not to influence further measurements).

The minimum applied load was 20 g; for smaller values, no appreciable spring displacement was noticed; the upper limit was defined so that the NiTi springs are not damaged (i.e., by applying loads higher than 200 g, the springs may undergo a permanent elongation); the load increment was set to 20 g to collect a significant amount of data. The process was repeated three times per each applied weight, in order to ensure the necessary repeatability of the measurements.

### 3.2. Results and Observations

**Figure 9:**
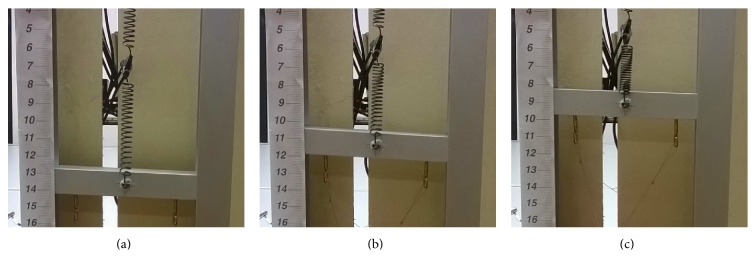
Test I: zero twist proof, both springs stimulated: (a) initial configuration; (b) spring contraction; (c) spring at its final minimum length. No angular displacement of the perversion has been observed during the motion.

Observations made during test I experiments, concerning the motion of the prototype, lead to some considerations on the crucial importance of the perversion. When a single spring lifts a load, a torque is experienced, while by connecting two springs with opposite rotational directions, two opposite torques are created and they cancel one another out. The presence of the perversion therefore imposes an almost pure linear movement of the tendril body when lifting loads. Indeed, when the series of springs was actually stimulated, they contracted and the load was lifted showing no appreciable rotational movement, confirming the predictions of the simulations and the expected behavior (Figure 9).

**Figure 10:**
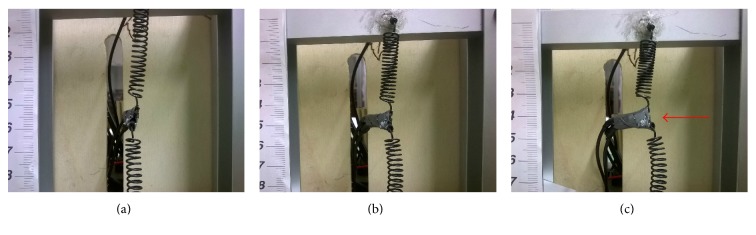
Test I: twisting effect, one spring only stimulated: (a) initial configuration; (b) the spring is driven and it starts rotating while lifting the weight; (c) at a visual inspection, the perversion in its final position has experienced a rotation of about 80°, as indicated by the red arrow.

As a further proof of this statement, only the upper spring was stimulated electrically. The progression of the “tendril” rotation was of about 80°, while lifting the weight upwards during contraction (Figure 10), showing how the torsional effect would occur without a counteracting torque.

**Figure 11:**
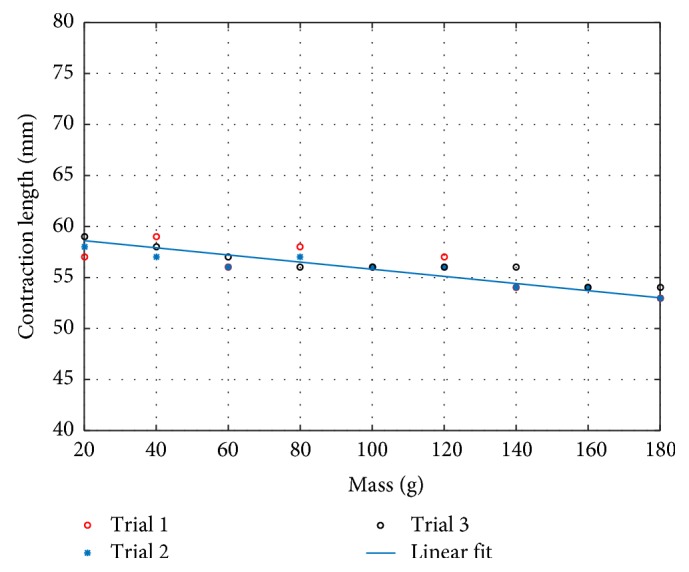
Linear regression of load-displacement experimental data (the different shaped dots represent the results of three repetitions of the test).

The results of test II are presented in Table 4, where the contraction displacement of the SMA spring system in relation to the applied load for all three test repetitions is reported. By computing the relationship between the applied force and the length variation of the tendril body, an almost linear relationship was found, which is convenient for controlling the actuator. As mentioned in Section 3.1, the upper limit of the load applied is 200 g; hence, the 180 g was the highest load used for presenting the linear relationship.  Figure 11 shows a graphical representation of the experimental data of Table 4 and of the system stiffness.

The best-fit linear regression of the collected data is calculated as follows:

(2) $$\begin{array}{c} d\left(x\right)=-0.035x+59.311, \end{array}$$

where *d* is the contraction length in millimeters and *x* is the applied weight in grams.

## 4. Conclusions

The free-coiling mechanism of a tendril-bearer climbing plant was investigated, and subsequently, the occurring geometrical mutation was replicated and characterized. The findings were used to devise a conceptual linear actuator. Previous theoretical studies on the nature of tendrils [8, 17] were reconsidered and extended by means of FE analyses of the tendril behavior. Investigations about the specific biological structure of the plant as well as the analysis of the effects of its peculiar degrees of freedoms were fundamental to set up the numerical models. More in detail, starting from the first modeling of the G-cell free-coiling, the simulation evolved into the emulation of the free coiling of the entire tendril body.

Even though the properties assigned to the G-cells as well as to the elastic layer were not faithfully representing the biological behavior of the tendril's internal structure, nevertheless, the model provided a successful description of the motion during the free-coiling phase. A further numerical study explored the conceptual possibility of realizing a linear actuator based on the same biomechanisms. The research then focused on the reproduction of a real conceptual model. With the aid of SMAs, the process of lifting a weight using free coiling was replicated in lab, realizing an equivalent “mechanical” tendril. The relation between contraction length of the system and the applied force was found to maintain an almost linear relationship. Furthermore, the influence of the perversion on the tendril motion was revealed and proved: it ensures that the tendril-like system experiences no torque while uplifting loads.

## Figures and Tables

**Table 1:** List of parameters used to define the G-cell model.

Parameter	Symbol	Value
Total length	*l*	60 mm
Total thickness	*t*	0.3 mm
Total height	*h*	0.9 mm
Spring constant	*k*	0.5 N/mm
*Material 1*		
Young's modulus	*E*	200000 N/mm^2^
Poisson's ratio	*v*	0.3
Thermal expansion coefficient	*α*	0.0075 mm/mmK
Thermal conductivity	*λ*	0.0054 W/mmK
Yield stress	*σ*	360 Mpa
Tangent modulus	*T*	2000 Mpa
*Material 2*		
Young's modulus	*E*	120000 N/mm^2^
Poisson's ratio	*v*	0.3
Thermal expansion coefficient	*α*	0.0075 mm/mmK
Thermal conductivity	*λ*	0.0054 W/mmK
Yield stress	*σ*	180 Mpa
Tangent modulus	*T*	1500 Mpa

**Table 2:** List of parameters of the entire tendril body model.

Parameter	Symbol	Value
Total length	*l*	60 mm
Total thickness	*t*	0.3 mm
Total height	*h*	0.9 mm
Spring constant	*k*	0.5 N/mm
*Material 1*		
Young's modulus	*E*	200000 N/mm^2^
Poisson's ratio	*v*	0.3
Thermal expansion coefficient	*α*	0.0015 mm/mmK
Thermal conductivity	*λ*	0.0054 W/mmK
Yield stress	*σ*	360 Mpa
Tangent modulus	*T*	2000 MPa
*Material 2*		
Young's modulus	*E*	120000 N/mm^2^
Poisson's ratio	*v*	0.3
Thermal expansion coefficient	*α*	0.0015 mm/mmK
Thermal conductivity	*λ*	0.0054 W/mmK
Yield stress	*σ*	180 MPa
Tangent modulus	*T*	1500 MPa
*Material 3*		
Mooney-Rivlin hyperelastic table	C10	0.0275
	C01	0.13077
	C11	0.68026
	*d*	0.048694

**Table 3:** List of parameters of the linear actuator model.

Parameter	Symbol	Value
Total length	*l*	60 mm
Total thickness	*t*	0.3 mm
Total height	*h*	0.9 mm
Spring constant	*k*	3 N/mm
*Material 1*		
Young's modulus	*E*	200000 N/mm^2^
Poisson's ratio	*v*	0.3
Thermal expansion coefficient	*α*	0.0075 mm/mmK
Thermal conductivity	*λ*	0.0054 W/mmK
Yield stress	*σ*	360 MPa
Tangent modulus	*T*	2000 Mpa
*Material 2*		
Young's modulus	*E*	120000 N/mm^2^
Poisson's ratio	*v*	0.3
Thermal expansion coefficient	*α*	0.0075 mm/mmK
Thermal conductivity	*λ*	0.0054 W/mmK
Yield stress	*σ*	180 MPa
Tangent modulus	*T*	1500 MPa

**Table 4:** Experimental load-displacement data.

Applied weight (g)	Contraction length (mm)		
	Trial 1	Trial 2	Trial 3
20	58	57	59
40	57	59	58
60	56	56	57
80	57	58	56
100	56	56	56
120	56	57	56
140	54	54	56
160	54	54	54
180	53	53	54
200	50	50	51
